# Optical imaging of MMP-12 active form in inflammation and aneurysm

**DOI:** 10.1038/srep38345

**Published:** 2016-12-05

**Authors:** Mahmoud Razavian, Thomas Bordenave, Dimitris Georgiadis, Fabrice Beau, Jiasheng Zhang, Reza Golestani, Jakub Toczek, Jae-Joon Jung, Yunpeng Ye, Hye-Yeong Kim, Jinah Han, Vincent Dive, Laurent Devel, Mehran M. Sadeghi

**Affiliations:** 1 Cardiovascular Molecular Imaging Laboratory, Section of Cardiovascular Medicine and Yale Cardiovascular Research Center, Yale University School of Medicine, New Haven, CT USA; 2 Veterans Affairs Connecticut Healthcare System, West Haven, CT USA; 3 Commissariat à l’Energie Atomique, iBiTec-S, Service d’Ingénierie Moléculaire de Protéines, CE-Saclay, 91191 Gif –sur-Yvette cedex, France; 4 Department of Chemistry, Laboratory of Organic Chemistry, University of Athens, Panepistimiopolis, Zografou, 15771, Athens, Greece

## Abstract

Matrix metalloproteinase (MMP)-12 plays a key role in the development of aneurysm. Like other members of MMP family, MMP-12 is produced as a proenzyme, mainly by macrophages, and undergoes proteolytic activation to generate an active form. Accordingly, molecular imaging of the MMP-12 active form can inform of the pathogenic process in aneurysm. Here, we developed a novel family of fluorescent probes based on a selective MMP-12 inhibitor, RXP470.1 to target the active form of MMP-12. These probes were stable in complex media and retained the high affinity and selectivity of RXP470.1 for MMP-12. Amongst these, probe **3** containing a zwitterionic fluorophore, ZW800-1, combined a favorable affinity profile toward MMP-12 and faster blood clearance. *In vivo* binding of probe **3** was observed in murine models of sterile inflammation and carotid aneurysm. Binding specificity was demonstrated using a non-binding homolog. Co-immunostaining localized MMP-12 probe binding to MMP-12 positive areas and F4/80 positive macrophages in aneurysm. In conclusion, the active form of MMP-12 can be detected by optical imaging using RXP470.1-based probes. This is a valuable adjunct for pathophysiology research, drug development, and potentially clinical applications.

Matrix metalloproteinases (MMPs) are a family of zinc-dependent endopeptidases which play important roles in tissue inflammation and remodeling, in part through cleavage of matrix proteins and other substrates^1^. Characteristically, these secreted, transmembrane, or plasma membrane-anchored proteins are biosynthesized as proenzymes which can be activated to expose an active catalytic site. MMP-12, a 55 kDa protein best known for elastolytic activity of its active form, is upregulated in aneurysm, atherosclerosis, cancer, chronic obstructive pulmonary disease and rheumatoid arthritis and may be a therapeutic target for these diseases^2^,^3^,^4^. In conjunction with its role in matrix remodeling, MMP-12 expression is closely linked to tissue inflammation. Macrophages are the major sources of MMP-12^4^,^5^,^6^ and notably, MMP-12 expression has been linked to alternative (M2) activation^7^,^8^. MMP-12 regulates inflammatory cell trafficking^9^ and its active form is retained at the cell membrane of macrophages^10^. Recently, MMP-12 has emerged as a regulator of gene transcription that plays a role in anti-viral immunity^5^.

The roles of MMPs in cardiovascular, pulmonary and other pathologies have led to the development of a number of non-selective tracers for detecting MMPs or their activity *in vivo*. While promising, molecular imaging based on such non-selective tracers can be confounded by the diversity of MMP effects^11^. Addressing whether panMMP imaging or targeting a specific member of MMP family is more effective in any specific setting is hampered by lack of highly selective MMP tracers. Given the role of MMP-12 in aneurysm^12^,^13^,^14^,^15^, MMP-12 imaging may provide unique information on the pathogenic process in this disease. To this end, we developed a novel family of fluorescent probes based on RXP470.1, a highly specific MMP-12 inhibitor^16^. These probes showed high affinity and selectivity for MMP-12 *in vitro*. The most promising probe was further evaluated for imaging of the active form of MMP-12 in murine models of sterile inflammation and aneurysm.

## Results

### Design and *in vitro* characterization of MMP-12 probes

**Figure 1:**
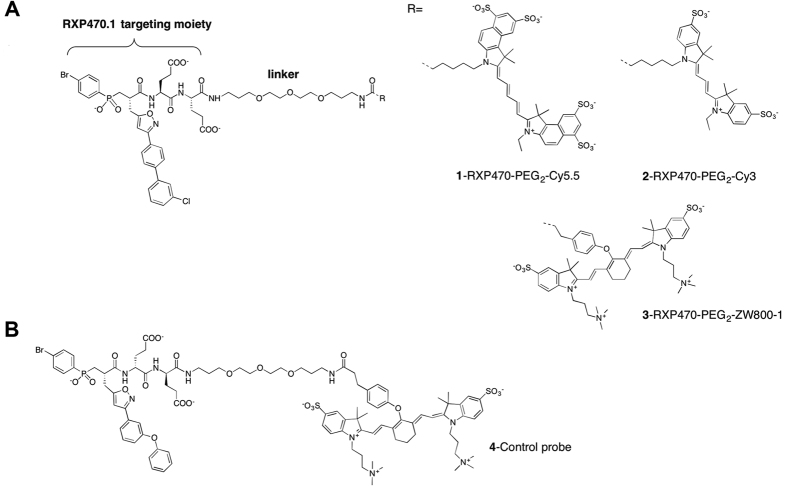
Near infrared fluorescent probes for targeting the active form of MMP-12. (**A**) Structures of MMP-12 targeting probes incorporating a polyethylene glycol (PEG) and a fluorescent dye R: Cy5.5, Cy3 and ZW800-1 respectively for probes 1, 2 and 3. (**B**) Structure of the control probe 4.

Based on crystal structure of the selective MMP-12 inhibitor, RXP470.1 in complex with the catalytic domain of human MMP-12, we designed and synthesized three fluorescent probes: a Cy5.5- conjugated probe **1** (global net charge = −6), a Cy3-labeled probe **2** and ZW800-1-labeled probe **3** (global net charge = −2) (Fig. 1 and Supplemental Table 1). The identity and purity of probes were confirmed by high performance liquid chromatography (HPLC), mass spectrometry and nuclear magnetic resonance spectroscopy (NMR). Stability evaluation of probe **3** showed that it is fully stable in PBS (data not shown) and in mouse blood over a period of 4 hours at 37 °C (Supplemental Fig. 1).

**Figure 2:**
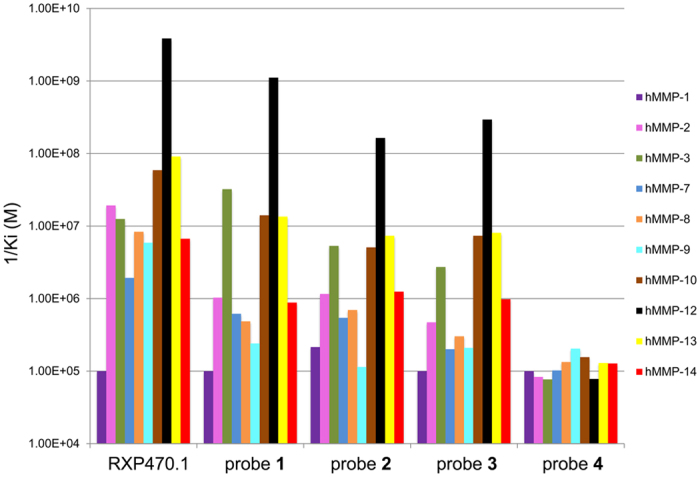
Comparison of affinity and selectivity profiles between RXP470.1 and probes **1** to **4** toward a panel of human (h) MMPs.

The affinity and selectivity profiles of the RXP470.1-derived probes were determined *in vitro* towards a set of 10 human MMPs (Fig. 2 and Supplemental Table 2). In comparison with RXP470.1, the addition of a short linker and a Cy5.5 dye (probe **1**) moderately impacted the affinity constant toward MMP-12 (0.26 nM *vs* 0.90 nM). In parallel, this structural modification resulted in a loss of potency toward other MMPs, with the exception of MMP-3, ranging from less than ten times in the case of MMP-7, -9, -10, or -13 to 30 times for MMP-8. Interestingly, the chemical nature of the fluorescent dye modestly impacted the affinity profile of the probes. Accordingly, both probe **2** with a Cy3 and probe **3** with a zwitterionic fluorophore, ZW800-1, remained potent and selective towards MMP-12 (Fig. 2 and Supplemental Table 2).

### Pharmacokinetics and biodistribution

**Figure 3:**
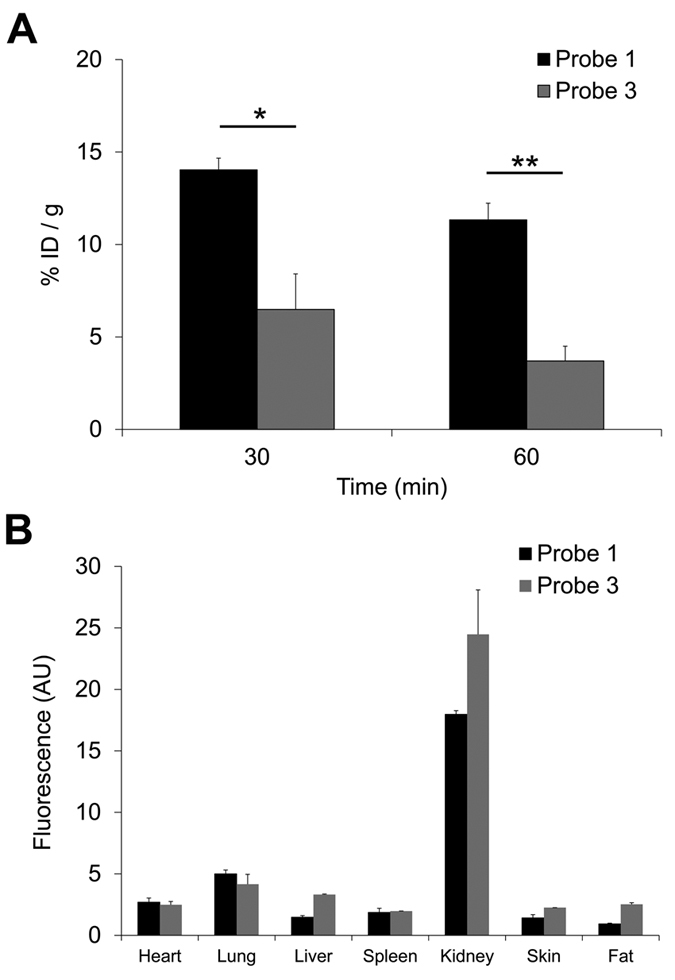
Blood levels (**A**) and biodistribution at 60 minutes after intravenous administration (**B**) of probes 1 and 3. n = 3 for each group. *p < 0.05, **p < 0.01. P: probe, ID: injected dose, AU: arbitrary units.

The blood clearance and biodistribution of MMP-12-targeted near infrared fluorescent probes were evaluated following intravenous administration (1 nmol) in wild type mice. As shown in Fig. 3A, the ZW800-1-conjugated probe **3** showed a significantly lower residual blood level compared to its Cy5.5-conjugated homolog, probe **1**, at both 30 and 60 minutes (p < 0.05 and <0.01, respectively for 30 and 60 minutes, n = 3 in each group). Evaluation of tissue fluorescence (using appropriate excitation and emission wavelengths) in organs harvested at 60 minutes showed considerably higher fluorescence signal in the kidneys relative to the liver, indicating renal clearance of the probes (Fig. 3B). We selected probe **3** for further evaluation based on its blood clearance profile.

### Sponge model of sterile inflammation

**Figure 4:**
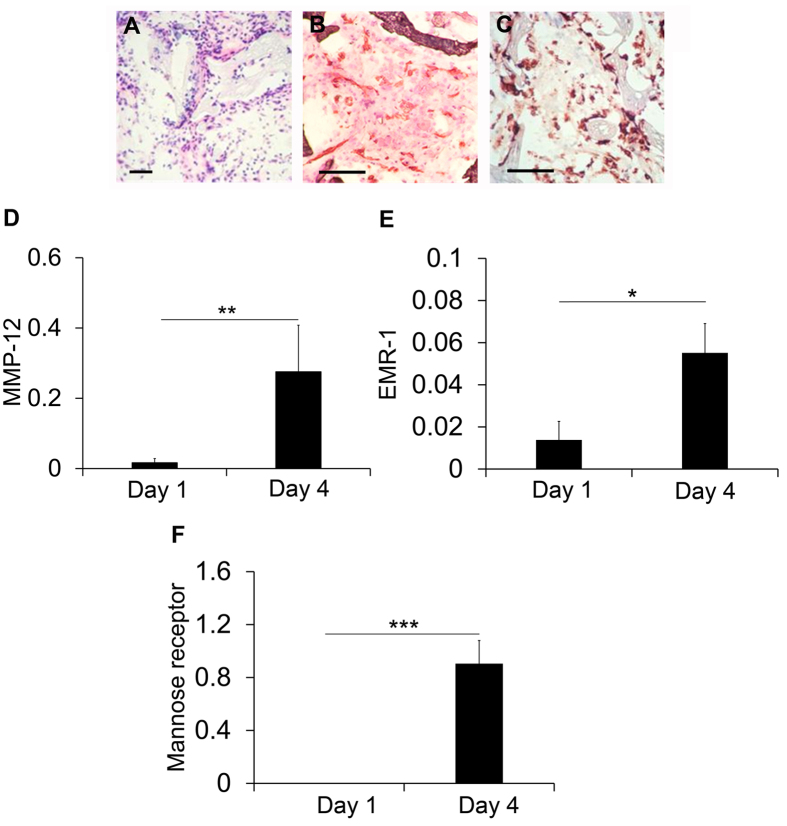
Sponge-implantation mouse model of sterile inflammation. (**A–C**) Representative H & E staining (**A**), and CD31 (**B**) and CD68 (**C**) immunostaining of sponge tissues harvested at day 4 after implantation, demonstrating tissue cellularization with considerable angiogenesis and inflammation. Scale bar = 200 μm. (**D–F**) Quantitative RT-PCR-derived analysis of MMP-12 (**D**), EMR1 (**E**), and mannose receptor (**F**) expression (normalized to GAPDH) in sponge tissues harvested at day 1 and day 4 after implantation. n = 3–8, *p < 0.05, **p < 0.01, ***p < 0.001.

As a prelude to evaluation of MMP-12 probes in clinically relevant models of cardiovascular pathologies, we focused on a model of sterile inflammation^17^. MMP expression and inflammation were assessed at 1 and 4 days after subcutaneous implantation of pieces of synthetic sponge in C57BL/6 mice. Consistent with previous reports^17^, we found considerable cell infiltration (Fig. 4A), angiogenesis (Fig. 4B) and inflammation (Fig. 4C) in sponges harvested 4 days after implantation. MMP-12 expression increased and MMP-9 decreased from day 1 to day 4 after implantation, while there was no clear difference in MMP-2 expression during this period (Fig. 4D and Supplemental Fig. 2). In parallel, EMR1, a marker of macrophages, and mannose receptor, a marker of alternatively polarized macrophages which express high levels of MMP-12, were upregulated in explanted sponges at 4 days (Fig. 4E,F), establishing this as a suitable model for MMP-12 binding studies.

### *In vivo* uptake and specificity

**Figure 5:**
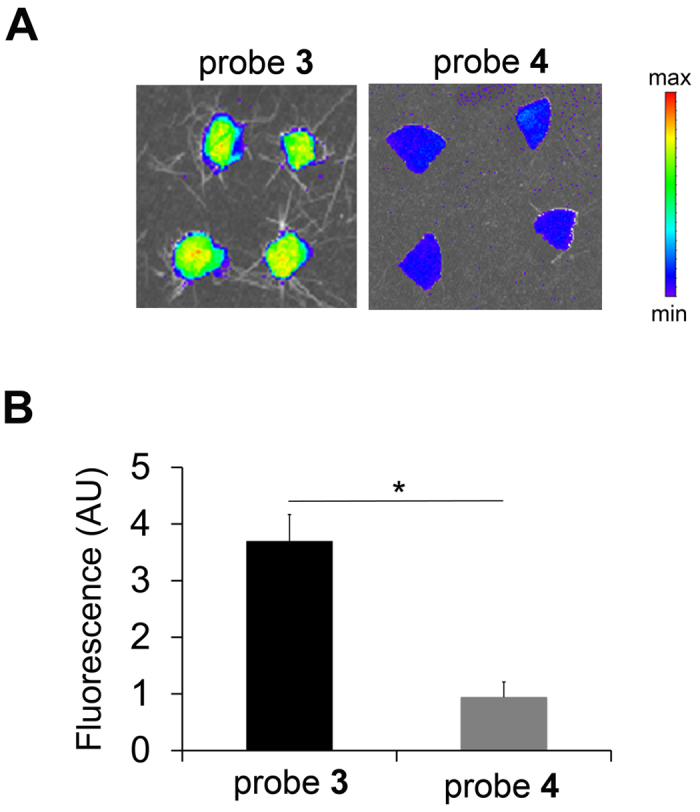
*In vivo* tissue binding and specificity of probe 3. Representative fluorescent images (**A**) and quantitative analysis of fluorescent signal (**B**) of the sponges explanted at 1 h after intravenous administration of probe **3** (n = 7) or **4** (n = 2), *p < 0.05. P: probe. AU: arbitrary units.

*In vivo* binding of probe **3** was first explored in the aforementioned sponge model of sterile inflammation. Near-infrared fluorescence reflectance imaging of sponges explanted 60 minutes after intravenous administration of probe **3** (1 nmol) showed an intense signal in the sponge (Fig. 5A). To address the *in vivo* binding specificity of probe **3**, a structurally similar, non-binding probe (probe **4)**, was developed (Fig. 1, see supplement information for details). This probe showed no affinity for human MMPs (Fig. 2) and was stable in mouse blood for 4 hours at 37 °C (Supplemental Fig. 1). Following intravenous administration of probe **4** (1 nmol), the fluorescence signal in sponges harvested at 60 minutes was significantly lower than the signal detected in sponges harvested at a similar time point from mice injected with probe **3** (Fig. 5B, n = 7 and 2 respectively for probes **3** and **4**, p < 0.05), supporting the *in vivo* binding specificity of probe **3**.

### MMP-12 imaging of carotid aneurysm

**Figure 6:**
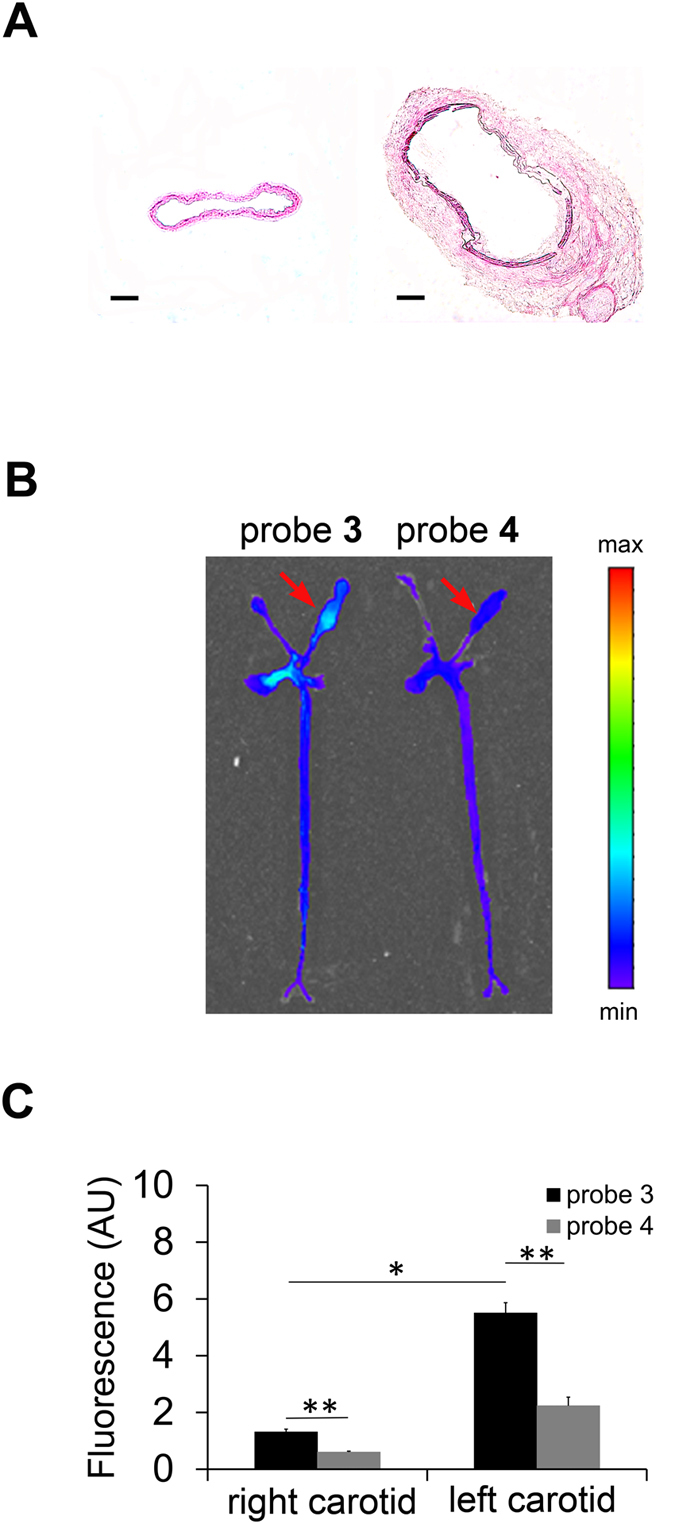
MMP-12 imaging of carotid aneurysm in apoE^−/−^ mice. (**A**) Representative H&E staining of NaCl-exposed right (left image) and CaCl_2_-exposed left (right image) carotid arteries in apoE^−/−^ mice at 4 weeks after surgery to induce aneurysm (scale bar 100 μm). (**B,C**) Representative fluorescent images (**B**) and quantitative analysis of fluorescent signal (**C**) from aortae and carotid arteries harvested at 1 h after intravenous administration of probe **3** (n = 3) or **4** (n = 4). *p < 0.01, **p < 0.001. Arrows point to aneurysmal left carotid artery. AU: arbitrary units.

Next, we investigated the feasibility of MMP-12-targeted imaging in a model of carotid artery aneurysm in high fat-fed apoE^−/−^ mice, where adventitial application of CaCl_2_ to carotid artery leads to significant MMP-12 upregulation, inflammation, and carotid artery dilatation (Fig. 6A) after 4 weeks^18^. In line with significant upregulation of MMP-12 in aneurysm, in animals injected with probe **3** the fluorescence signal in the aneurysmal left carotid artery was significantly higher than the signal in the control, contralateral artery in tissues harvested at 60 minutes (Fig. 6B and C, n = 3, p < 0.01). In animals injected with the non-binding probe **4,** fluorescence imaging of carotid arteries harvested at 60 minutes showed a significantly lower signal in the carotid aneurysm compared to animals injected with probe **3**, establishing the specificity of probe **3** signal *in vivo* (Fig. 6B and C, n = 3 and 4, respectively for probes **3** and **4**, p < 0.001). Notably, the residual blood levels of probes **3** and **4** were comparable at both 30 and 60 minutes post-injection (Supplemental Table 3) and morphometric analysis of carotids showed no difference in aneurysm size between the two groups of animals (data not shown).

**Figure 7:**
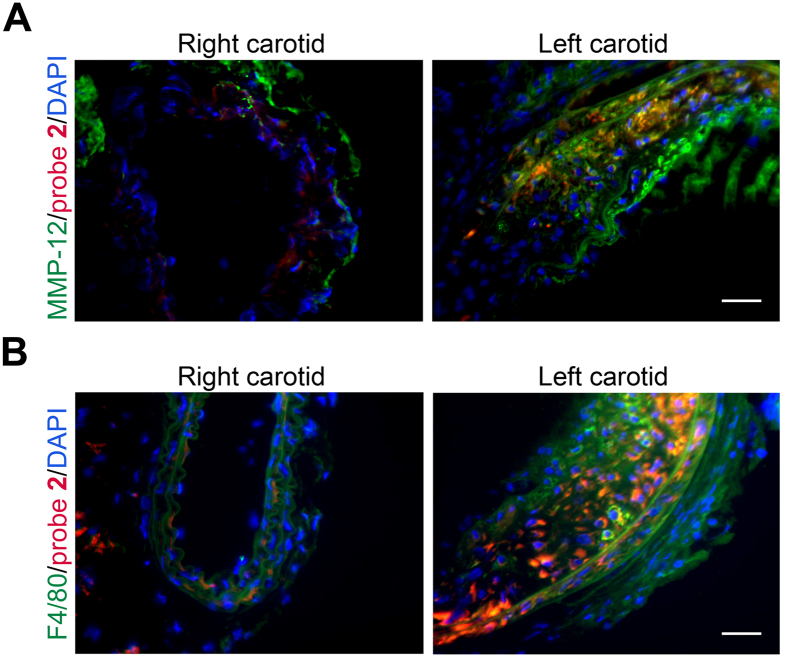
MMP-12 probe binding and localization in murine carotid arteries. Representative examples of carotid artery co-immunostaining demonstrating co-localization of the Cy3-labeled probe **2** (in red) with MMP-12 (**A**) and F4/80 positive cells (**B**, both in green) in the aneurysmal left carotid artery. Nuclei are stained with DAPI in blue. Scale bar = 50 μm.

MMP-12 probe binding in aneurysm was further investigated by fluorescence microscopy using a RXP470.1-derived probe incorporating a Cy3 dye (probe **2**). In line with its specificity for the active form of MMP-12, the probe **2** signal partially co-localized with MMP-12 in the aneurysmal left carotid artery, while little MMP-12 or probe **2** binding could be detected in the control, right carotid artery (Fig. 7A). Similarly, probe **2** co-localized with F4/80 (a macrophage marker) positive cells in the aneurysmal carotid artery, but little F4/80 staining was detectable in the control carotid arteries (Fig. 7B).

## Discussion

Here we describe the development of the first MMP-12-targeted imaging probes and demonstrate their performance in murine models of sterile inflammation and aneurysm. MMP-12 plays an important role in the pathogenesis of aneurysm, atherosclerosis, chronic obstructive pulmonary disease and other pathologies and is under investigations as a therapeutic target^2^,^3^,^4^,^12^,^13^,^14^,^15^,^19^. As such, molecular imaging of MMP-12 can be a powerful tool for pathophysiology research, drug development and multiple clinical applications.

MMP-12 function is tightly regulated at the transcriptional and posttranscriptional levels. Like other MMPs, MMP-12 is produced as a pro-enzyme^1^. The activation of the precursor exposes a catalytic site which can be targeted for imaging. Similar to other MMPs, MMP-12 activity is a function of the enzyme’s expression level and presence of inhibitors. In addition, cellular localization appears to play an important role in MMP-12 functions. In cell culture studies, MMP-12 activity is observed near the surface of macrophages^10^, and the active form of MMP-12 binds to lipid bilayers and is quickly internalized to perinuclear structures, nuclear membrane and the nucleus^6^. The membrane-bound MMP-12 remains enzymatically active, but natural substrate accessibility (e.g., to bulky, rigid substrates) maybe selectively impaired^6^.

Not surprisingly, because of the potential clinical value of MMP-targeted imaging, a number of imaging agents have been developed. These agents can be categorized into two classes: functionalized inhibitors, which bind directly to an activated enzyme, and activatable probes that generate a signal following proteolytic cleavage of a substrate^20^. Of those MMP imaging probes evaluated in animal models of cancer and cardiovascular disease, only a few have shown a specific signal and potential for further development^20^. The most promising agents include the radiolabeled macrocyclic agents, RP782 and RP805. SPECT studies based on these panMMP-specific agents detect MMP active forms in ventricular and vascular remodeling and can be used to predict aneurysm rupture in the mouse^21^,^22^,^23^. MMP signal often correlates with tissue inflammation and is reduced in response to therapeutic interventions^24^,^25^,^26^. Given the diverse, and at time opposite roles that MMPs play in cardiovascular pathology, imaging a specific member of MMP family could provide additional information regarding specific cardiovascular pathologies.

Our novel MMP-12 specific probes are derived from a highly potent and selective MMP-12 inhibitor, the phosphinic pseudo-peptide, RXP470.1^16^. In high fat-fed apoE^−/−^ mice, RXP470.1 reduces atherosclerotic plaque area and promotes a fibrous plaque phenotype^3^. More recently, this compound was also shown to block MMP-12 activity in mouse models of inflammation^27^,^28^ and during viral infection^5^. The similarity between the effects of RXP470.1 and MMP-12 gene deletion in models of atherosclerosis and viral infection^3^,^5^ suggests that MMP-12 active form is the privileged target for this inhibitor *in vivo*. Based on these observations, we predicted that RXP470.1 would be an appropriate starting point for developing MMP-12 specific probes. The rational design of such probes relied on the 3D-structure analysis of RXP470.1 in interaction with MMP-12 catalytic domain^29^. This indicated that a chemical elongation on the *C*-terminal carboxamide function would be appropriate for installing a fluorescent reporter. The impact of the fluorophore structure and the net charge on *in vitro* and *in vivo* properties of the tracers was assessed by comparing two near infrared dyes, Cy5.5 and ZW800-1. In other series of probes based on small ligands, these issues were found to be critical, especially regarding the tracers’ *in vivo* performance^30^,^31^.

The optical probes in our study consist of two chemical entities of comparable size, a small pseudo peptide as targeting moiety, and a symmetric indocarbocyanine as reporter dye. In such a situation, the properties of the dye (e.g. chemical structure and global net charge, see Fig. 1 and Supplemental Table 1) and how it is connected to the recognition element can significantly affect the probe binding properties towards its privileged target. Very recently, the crystal structure of probe **1** in complex with the MMP-12 catalytic domain was obtained^32^. This structure reveals both that RXP470-derived probe **1** preserves all the interactions established by its parent molecule and that the fluorophore moiety tethered to the polyethylene spacer (PEG_2_) extends beyond the S_3_’ region. In solution, these observations are corroborated by affinity measurements where probe **1** exhibits a comparable affinity for MMP-12 as RXP470.1 (Fig. 2 and Supplemental Table 2). By assuming that probes **2** and **3** adopt a similar binding mode to that of probe **1**, the small differences in potency towards MMP-12 between the three probes (factors 7 and 4 relative to probe **1** for probes **2** and **3,** respectively) can be ascribed to the dye nature itself. Particularly, the negatively charged Cy5.5 dye (probe **1**, Supplemental Table 1) seems to be more tolerated within the MMP-12 catalytic domain than the ZW800.1 moiety with a positive net charge (probe **3**, Supplemental Table 1). This observation is consistent with the presence of several basic residues at the MMP-12 surface. In addition, other interactions between the fluorescent group and residues from the S_1_’ loop cannot be excluded. However, due to the high flexibility of the PEG_2_ spacer in solution, such interactions remains difficult to predict. Regarding the other MMPs, with the exception of MMP-3, all the probes share a similar drop in affinity from a factor 3 (probe **1** towards MMP-7) to a factor 50 (probe **2** towards MMP-9), as compared to the RXP470.1 inhibitor. In term of energy of binding, these variations correspond to rather weak differences (from 0.6 to 2.3 kcal/mol) that may be explained, at least in part, by the structural differences between the MMPs particularly in their S_1_’ loops^33^,^34^,^35^,^36^,^37^. Thus due to the high flexibility of the PEG_2_ spacer in solution, a particular fluorophore may have interactions with S_1_’ loop residues for one MMP, which would not occur with another MMP. This would lead to the observed variations in the probe selectivity profile according to the nature of the fluorescent reporter. In solution, other factors such as differences in desolvation energy between the three probes and the parent RXP470.1 may also contribute to variances in their energy of binding. Overall, in this series of tracers, the MMP binding profile is mainly ruled by the RXP470.1 motif.

The dye structure and net charge appear to have a major effect on blood clearance, with the ZW800-1 conjugated probe (probes **3**) exhibiting significantly lower residual blood level at 60 minutes compared to its Cy5.5-conjugated homolog (probe **1**). The *in vivo* performance of ZW800-1 itself is remarkable by a rapid renal clearance^38^. Surprisingly, only a few studies discuss the influence of ZW800-1 on blood clearance when conjugated to small ligands^39^. Our study demonstrates that ZW800-1 conjugation to RXP470.1 induces blood clearance acceleration in comparison with Cy5.5 conjugation. Accordingly, ZW800-1 seems to be suitable for generating RXP470-derived fluorescent probes with short *in vivo* half-life. Given the juxtaposition of the vessel wall and blood, the signal emanating from the blood can interfere with vascular molecular imaging studies. Therefore, probe **3** was selected for further *in vivo* evaluation.

Animal models of cardiovascular pathology are generally based on an inflammatory response to mechanical or other types of injury and several weeks are needed for the models to be fully developed. For our validation studies of probe **3** we relied on a sponge model of sterile inflammation, where considerable inflammation and tissue remodeling, as well as upregulation of MMP-12 can be induced within days of sponge implantation. The heterogeneity in inflammatory response was addressed by implanting multiple pieces of sponge in the same animal and averaging the data. We detected a significant difference in tracer accumulation in implanted sponges between probe **3** and its non-binding control, probe **4.** We confirmed these findings in a model of carotid aneurysm in high fat fed apoE^−/−^ mice, where we observed significantly higher signal in the aneurysmal carotid, compared to contralateral, control artery. In addition, there was uptake of the MMP-12 probe in aortic arch and innominate artery. These are areas of early atherosclerosis development in animals on high fat diet. While we have not analyzed these areas histologically, it is unlikely that any significant atherosclerotic plaque is present in the aortic arch of these animals only after 5 weeks of high fat diet. This raises the exciting possibility of a role for MMP-12 imaging in detecting the sites of early atherosclerosis prior to florid disease. Supporting signal specificity, neither the carotid nor aortic signal was present in animals injected with the non-binding, control probe **4**.

Given the role of MMP-12 in the pathogenesis of aneurysm^12^,^13^,^14^,^15^, MMP-12 imaging may be an effective tool for aortic aneurysm risk stratification. Indeed, many deaths in patients with abdominal aortic aneurysm occur in smaller aneurysms that do not meet the criteria for aneurysm repair. MMP imaging is emerging as a potentially promising approach for this purpose^23^,^40^. Addressing whether targeting a single MMP, e.g., MMP-12, is more effective than panMMP imaging for predicting aneurysm outcome remains to be empirically determined. Our fluorescent probes, which are potentially suitable for intravascular and intraoperative imaging applications^41^,^42^,^43^, are a step ahead in this direction. In parallel, based on the data obtained here radiolabeled homologs of probe **3**, which may be used for non-invasive imaging of MMP-12 active form in inflammation and tissue remodeling in a broad range of cardiovascular and other pathologies, are under development.

In conclusion, here we report the first selective probes for imaging the active form of macrophage elastase (MMP-12). These rationally designed probes are based on the structure of a selective, high affinity MMP-12 inhibitor, RXP470.1 and bind specifically to the sites of inflammation and tissue remodeling in preclinical murine models. Given the key role of MMP-12 in the pathogenesis of a broad range of cardiovascular, pulmonary and other pathologies, these probes should be valuable for pathophysiology research, drug development, and potentially clinical applications.

## Materials and Methods

### Reagents

ZW800-1 NHS ester^38^ was provided by Dr. John Frangioni (Flare foundation, http://www.theflarefoundation.org). All other reagents were purchased from Sigma Aldrich (Saint Louis, MO), unless otherwise specified.

### Synthesis, characterization and stability assessment of the fluorescent *probes*

Probes **1**, **2**, **3** and control probe **4** were synthesized from phosphinic building blocks whose synthesis was previously reported^16^. Briefly, the elongation of the peptide’s sequence as well as the incorporation of the phosphinic building blocks were performed on solid support using a standard Fmoc strategy. After cleavage from the support, pseudo peptide intermediates were purified and subsequently engaged in a coupling reaction with the appropriate dye-NHS ester. The identity and purity of each optical probe were confirmed by analytical HPLC, mass spectrometry analysis and NMR (for detailed protocols and analytical data see supplemental information). The stability of each probe was confirmed in phosphate buffered saline (PBS). Moreover, the stability of probe **3** and its control, probe **4** were evaluated by HPLC in mouse blood over a period of 4 hours at 37 °C.

### Affinity and selectivity profile

Human MMP-8, -9, -12 and -13 were produced in the laboratory^29^,^44^. Other MMPs were purchased from R&D Systems (Minneapolis, MN). MMP inhibition assays were carried out as previously described^16^. Pro-MMPs were pre-activated by *p*-aminophenylmercuric acetate following the method described by R&D Systems. Titration experiments were carried out to determine active enzyme concentration for each MMP prior to the assay^29^. For each probe, the percentage of inhibition was determined from five different concentrations in triplicates, chosen to reach a range of 20–80% inhibition. Ki values were determined using the method proposed by Horovitz and Leviski^45^.

### Animal models

Sponge model of sterile inflammation: Pieces of polyvinyl alcohol sponge (Ivalon Ear Wick, 0.9 e × 15 mm, New London, CT) were implanted in dorsal subcutaneous pockets of C57BL/6 mice (originally from Jackson Laboratory, Farmington, CT, n = 24) under isoflurane anesthesia, as described^17^. Animals were re-anesthetized at day 1 or 4 after sponge implantation, and the sponges were removed for imaging or embedded in OCT, snap-frozen, and stored at −80 °C for further analysis.

Carotid aneurysm model: Arterial aneurysm was induced over a 4-week period by exposing the left common carotid artery of apolipoprotein E–deficient (apoE^−/−^, n = 7) mice (Jackson Laboratory) to calcium chloride, as described^18^. The opposite carotid artery was exposed to normal saline and served as control for imaging studies. Tissues from a previous study (n = 3)^18^ supplemented histologic evaluations.

Buprenorphine (0.05 mg/kg, ip every 12 hours for 48 hours) was used for postoperative analgesia for both models. All experiments were carried out in accordance with the relevant guideline of, and protocols approved by Yale University and VA Connecticut Institutional Animal Care and Use Committees.

### Histology and immunostaining

Hematoxylin and eosin (H&E) staining and immunostaining of tissues were performed according to standard protocols on 5-μm-thick cryostat sections. For immunostaining, primary antibodies included anti-MMP-12 (sc-30072, Santa Cruz Biotechnology, Dallas, TX), anti-CD68 (AbD serotec, Raleigh, NC), anti-F4/80 (Cedarlane, Burlington, NC) and anti-CD31 (BD Pharmingen, San Jose, CA). Isotype-matched antibodies were used as controls. For co-immunostaining, tissue sections were fixed with acetone, and stained with the antibody followed by probe **2**. Nuclei were stained with DAPI using ProLong^®^ Gold Antifade Mountant with DAPI (Molecular Probes, Eugene, OR). The slides were photographed using a Spot RT3 camera (Diagnostic Instruments, Sterling Heights, MI) and a Zeiss Axiophot fluorescence microscope (Carl Zeiss Microscopy GmbH, Jena, Germany).

### Quantitative reverse-transcription polymerase chain reaction (RT-PCR)

RT-PCR was performed as described^46^ and the results were normalized to glyceraldehyde 3-phosphate dehydrogenase (GAPDH). The following TaqMan probe and primer sets (Applied Biosystems, Waltham, MA) were used: EMR1 (Mm00802529_m1), Mannose receptor (Mm01329362_m1), MMP-12 (Mm00500554_m1), and GAPDH (Mm99999915_g1).

### Western blotting

Protein samples were extracted from sponge implants (5-μm-thick cryostat sections) using RIPA buffer (Sigma, Saint Louis, MO) containing proteinase inhibitor cocktail (Roche Diagnostics, Mannheim, Germany). Samples were separated using 4–15% Mini-PROTEAN TGX precast gels (BioRad, Hercules, California). For Western blotting, the following antibodies were used: anti-MMP-2 (sc-13595, Santa Cruz Biotechnology, Dallas, Texas), anti-MMP-9 (AB19016, Millipore, Darmstadt, Germany), anti-MMP12 (AF3467, R&D systems, Minneapolis, MN), anti-actin (MAB1501, Millipore, Darmstadt, Germany).

### Near-infrared fluorescence reflectance imaging

Fluorescent probes (one nmol in 100 μL saline) were administered through a jugular vein catheter under inhaled isoflurane anesthesia. Blood samples were collected at various time points after probe administration to monitor blood clearance. Animals were sacrificed at the indicated time points and various organs were harvested. Near-infrared fluorescence reflectance imaging was conducted using an IVIS Spectrum system (Caliper Life Science, Hopkinton, MA) with appropriate excitation (Ex) and emission (Em) filter sets (Ex/Em = 675/720 nm for probe **1** and Ex/Em = 745/800 nm for probes **3** and **4**). Fluorescence from 10 μL of blood was converted to % injected dose per gram (% ID/g) using a standard curve derived from serial dilutions of each probe in 10 μL of mouse blood. Tissue fluorescence was quantified as mean efficiency per pixel and was presented as an arbitrary unit (AU) relative to the signal from each probe in 10 μL of mouse blood.

### Statistical analysis

Statistical analysis was performed by 2-tailed, unpaired or paired t-test, as indicated, using GraphPad Prism (version 6, La Jolla, CA). Data are presented as mean ± standard error (SE). Significance was set at the 0.05 level.

## Additional Information

**How to cite this article**: Razavian, M. *et al*. Optical imaging of MMP-12 active form in inflammation and aneurysm. *Sci. Rep.* **6**, 38345; doi: 10.1038/srep38345 (2016).

**Publisher's note:** Springer Nature remains neutral with regard to jurisdictional claims in published maps and institutional affiliations.

## Supplementary Material

Supplemental Data
