# Supplementary material for: Optical imaging of MMP-12 active form in inflammation and aneurysm
Source: Sci Rep. 2016 Dec 5;6:38345. doi: 10.1038/srep38345 (PMC5137160; doi:10.1038/srep38345)

Supplemental information

Optical imaging of MMP-12 active form in inflammation and aneurysm

Mahmoud Razavian, PhD[1, 2]*, Thomas Bordenave, PhD[3]*, Dimitris Georgiadis, PhD[4], Fabrice Beau[3], Jiasheng Zhang, MD[1, 2], Reza Golestani, MD, PhD[1, 2], Jakub Toczek, PhD[1, 2], Jae-Joon Jung, PhD[1, 2], Yunpeng Ye, PhD[1, 2], Hye-Yeong Kim, PhD[1, 2], Jinah Han, PhD[1, 2], Vincent Dive, PhD[3], Laurent Devel, PhD[3], Mehran M. Sadeghi, MD[1, 2]

1. Cardiovascular Molecular Imaging Laboratory, Section of Cardiovascular Medicine and Yale Cardiovascular Research Center, Yale University School of Medicine, New Haven, CT (USA); 2. Veterans Affairs Connecticut Healthcare System, West Haven, CT (USA); 3. Commissariat à l'Energie Atomique, iBiTec-S, Service d'Ingénierie Moléculaire de Protéines, CE-Saclay, 91191 Gif –sur-Yvette cedex, France, 4. Department of Chemistry, Laboratory of Organic Chemistry, University of Athens, Panepistimiopolis, Zografou, 15771, Athens, Greece

*: Equally contributed to this project

Address for correspondence:

Mehran M. Sadeghi, M.D.

Yale Cardiovascular Research Center

300 George Street, #770F

New Haven, CT 06511

Fax: 203-937 3884

Phone: 203-932 5711 x3398

mehran.sadeghi@yale.edu

Table of Contents

Supplemental experimental section……………………………..……………….…p3

General information……………………………..…………………….……..……..p3

*Synthesis of probes **1**, **2**, **3** and **4***……………………………………….....…....p4

*Stability of probes **3** and **4** in mouse blood*.……………………..……..…………p6

Supplemental Figure 1………..p7

Supplemental Figure 2………..p8

Supplemental Table 1………...p9

Supplemental Table 2………...p10

Supplemental Table 3………...p11

^{1}H NMR spectra of probes **1**, **2** and **3**……….…………………..……………..………p12

Supplemental experimental section

General information. All chemicals were commercial products of analytical grade and were used without further purification unless otherwise specified. Universal polyethylene glycol (PEG) NovaTagTM resin (code: 855058, substitution: 0.24 mmol/g), Fmoc-(L)-Glu(OtBu)-OH (code: 852009) and Fmoc-(D)-Glu(OtBu)-OH (code: 852155) were purchased from Merck Millipore (Darmstadt, Germany). Cy®3 Mono-reactive-NHS ester and Cy®5.5 Mono NHS ester were purchased from Sigma Aldrich (Saint Louis, MO, USA, code: GEPA13105, and code: GEPA15601 respectively). ZW800-1 NHS ester was provided by Dr. John Frangioni (Flare foundation, http://www.theflarefoundation.org). All other reagents were purchased from Sigma Aldrich (Saint Louis, MO, USA). Pseudo-peptide synthesis was performed manually in polypropylene syringe equipped with a polyethylene frit and a stopper. Microwave experiments were performed on a Discover apparatus (CEM µWave, Matthews, NC, USA) using open vessel mode with SPS kit. ^{1}H NMR spectra of probes **1**, **2** and **3**, were recorded on a Bruker Avance 600 MHz with a TCI CryoProbe. Compound analysis by Reversed Phase High Pressure Liquid Chromatography (RP-HPLC) was performed on Supelco Ascentis® Express C18 column (100 × 4.6 mm, 2.7 µm, flow rate = 1.2 mL/min), Grace Vision HT HL C18 column (150 × 4.6 mm, 3 µm, flow rate = 1.2 mL/min) or Agilent XDB C18 column (150 × 4.6 mm, 5 µm, flow rate = 0.6 mL/min). Compound purification was performed on Supelco Ascentis® C18 column (150 × 10 mm, 5 µm, flow rate = 3 mL/min). UV detection was performed at 230 nm, 280 nm or at the wavelength corresponding to the maximum of fluorochrome absorption. A solvent system consisting of (A) 0.1% trifluoroacetic acid (TFA) in water and (B) 0.09% TFA in acetonitrile was used. Retention times (Rt) are reported in minutes. UV absorption and fluorescence measurements were performed on a UV-1800 spectrophotometer (Shimadzu, Kyoto Japan), and on Cary Eclipse apparatus (Varian, Palo Alto, CA), respectively. Mass spectrometry data were collected using a 4800 MALDI-TOF mass spectrometer (Applied Biosystems, Foster City, CA) or an ESI ion trap Esquire HCT spectrometer (Bruker Daltonics, Billerica, MA). Amino acid compositions were characterized using an AminoTac JLC-500/V amino acid analyzer (JEOL, Tokyo, Japan). The identity and purity of each synthesized compound were assessed by NMR, analytical HPLC (two conditions of elution) and mass spectrometry. Fluorescence measurements were

performed at room temperature. For each pseudo-peptide, the molar extinction coefficient was calculated from the absorbance value of a solution whose concentration was first determined by amino acid composition.

Synthesis of probes ***1****,* ***2****,* ***3*** *and* ***4*****:** The probes were synthesized as illustrated below:

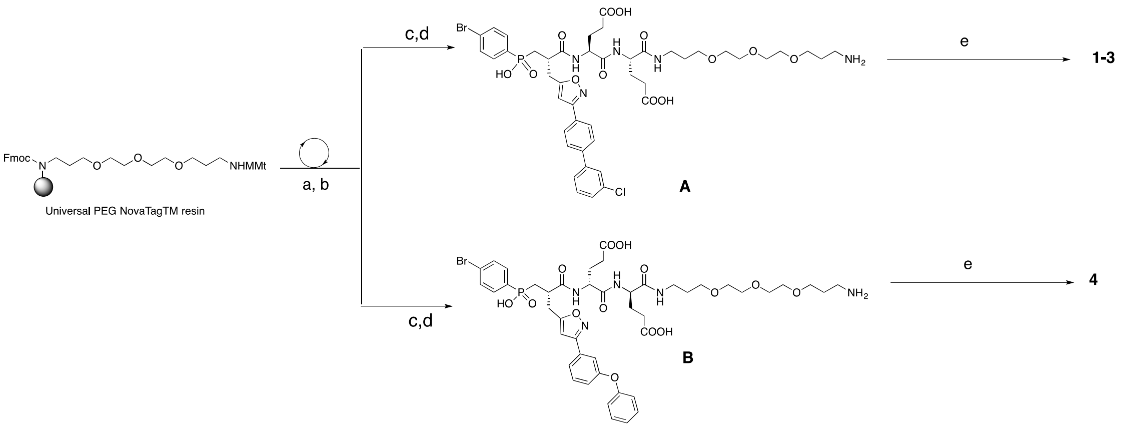

Reagents and conditions: (a) Fmoc solid phase synthesis, Fmoc-(L)-Glu(OtBu)-OH or Fmoc-(D)-Glu(OtBu)-OH (10 eq), ClHOBt/DIC (10 eq), DMF; 60°C, microwave irradiation at 45W, 10 min, (b) Phosphinic block (1.2 eq), ClHOBt/DIC (3 eq), DMF; 60°C, microwave irradiation at 45W, 60 min, (c) TFA/TIS/H_2O: 95/2.5/2.5, RT, 3x45 min, (d) RP-HPLC separation (e) NIR dye NHS ester (1.5 eq), DIEA (10 eq), DMF, RT, ON followed by HPLC purification.

Briefly, standard Fmoc methodology was used to build the amino acid sequence on Universal PEG NovaTag^{TM} resin. Fmoc-(L)-Glu(OtBu)-OH or Fmoc-(D)-Glu(OtBu)-OH (10 equivalents) and appropriate phosphinic building blocks (1.2 equivalents) were incorporated on a solid support following a standard protocol in the presence of N,N'-diisopropylcarbodiimide (DIC) and 6-chloro-1-hydroxybenzotriazole di-hydrate (ClHOBt) in dimethylformamide (DMF). The coupling reactions for amino acid and phosphinic building blocks were performed under microwave irradiation (45 W) at 60°C for 10 min and 60 min, respectively. After cleavage from the support with TFA/triisopropylsilane/water 95:2.5:2.5 cocktail (5 mL, 3 × 45 min) and with TFA/DCM solution 1:1 (5 mL, 2 × 45 min), the crude pseudo peptides, a mixture of diastereomers, were purified by RP-HPLC on Supelco Ascentis® C18 column (0 to 100% B in 30 min) to afford intermediate **A** (17% yield) and **B** (24% yield) as white powders after freeze drying. To a solution of **A** or **B** (1 equivalent) in DMF (C = 2 mM) were added successively *N,N*-diisopropylethylamine (10 equivalents) and a solution of the appropriate NHS-activated fluorescent dye in DMF (1.5 equivalents, C = 3 mM). The resulting solution was stirred at room temperature and the reaction progress was monitored by RP-HPLC (Supelco Ascentis® Express C18 column, 0 to

100% B in 15 min). The reaction mixture was quenched with water and the crude material was purified by RP-HPLC (Supelco Ascentis® C18 column, 0 to 100% B in 30 min). All synthesized fluorescent probes (**1-4**) were obtained as solids after freeze drying and stored at -20°C away from light.

Intermediate **A**: $\varepsilon_{272\ nm}$ = 33,550 $cm^{-1}M^{-1}$ in methanol; Rt = 6.28 min (Supelco Ascentis® Express C18 column, 0 to 100% B in 10 min) and Rt = 7.07 min (Grace Vision HT C18 HL column, 0 to 100%B in 10 min); ^1H NMR (600 MHz, d_6-DMSO) δ; 1.22 (s, 0.5H), 1.58 (m, 2H), 1.73 (m, 4H), 1.83-2.00 (m, 3H), 2.10-2.35 (m, 4H), 2.83 (m, 2H), 3.05 (m, 4H), 3.28-3.48 (m, 14H), 4.04 (m, 1H), 4.14 (m, 1H), 6.80 (s, 1H), 7.4-7.55 (m, 3H), 7.60-7.72 (m, 6H), 7.75-7.85 (m, 3H), 7.90 (m, 2H), 8.20 (bs, 1H), 8.40 (m, 1H); MS (MALDI-TOF) m/z for $[C_{45}H_{55}BrClN_5O_{13}P]^-$ calcd. 1018.2412, found 1018.3916.

Intermediate **B**: $\varepsilon_{230\ nm}$ = 38,000 $cm^{-1}M^{-1}$, $\varepsilon_{272\ nm}$ = 3,100 $cm^{-1}M^{-1}$ in Methanol; Rt = 6.09 min (Supelco Ascentis® Express C18 column, 0 to 100% B in 10 min) and Rt = 7.19 min (Grace Vision HT C18 HL column, 0 to 100% B in 10 min); MS (MALDI TOF) m/z for $[C_{45}H_{56}BrN_5O_{14}P]^-$ calcd. 1000.2750, found 1000.2074.

RXP470-PEG$_2$-Cy5.5, **1**. A blue solid was obtained (60%). In PBS: $\varepsilon_{278\ nm}$ = 42,600 $M^{-1}cm^{-1}$, $\varepsilon_{680\ nm}$ = 262,600 $M^{-1}cm^{-1}$; In PBS, $\lambda_{ex}/\lambda_{em}$ = 680/695 nm; Rt = 6.38 min (Supelco Ascentis® Express C18 column, 0 to 100% B in 10 min) and Rt = 6.55 min (Grace Vision HT C18 HL column, 0 to 100% B in 10 min); ^1H NMR (600 MHz, d_6-DMSO) δ; 1.24 (m, 5H), 1.36 (m, 5H), 1.54-1.59 (m, 5H), 1.76 (m, 4H), 1.96 (s, 12H), 2.05 (m, 3H), 2.12-2.21 (m, 6H), 3.04 (m, 8H), 4.07-4.16 (m, 3H), 4.23-4.29 (m, 3H), 6.36 (m, 3H), 6.64 (m, 1H), 6.79 (s, 1H), 7.46 (m, 2H), 7.52 (m, 3H), 7.63-7.84 (m, 8H), 7.91 (m, 2H), 8.12 (m, 2H), 8.20 (s, 1H), 8.39 (m, 1H), 8.45 (m, 3H), 9.02 (m, 2H); MS (MALDI-TOF) m/z for $[C_{86}H_{97}BrClN_7O_{26}PS_4]^-$ calcd. 1916.3981, found 1916.4143.

RXP470-PEG$_2$-Cy3, **2**. A pink solid was obtained (63%). In PBS: $\varepsilon_{278\ nm}$ = 42,200 $M^{-1}cm^{-1}$, $\varepsilon_{553\ nm}$ = 167,700 $M^{-1}cm^{-1}$; In PBS, $\lambda_{ex}/\lambda_{em}$ = 555/571 nm; Rt = 6.25 min (Supelco Ascentis® Express C18 column, 0 to 100% B in 10 min and Rt = 7.35 min (Grace Vision HT C18 HL column, 0 to 100% B in 10 min); ^1H NMR (600 MHz, d_6-DMSO) δ; 1.33 (m, 4H), 1.38 (m, 2H), 1.56 (m, 8H), 1.71 (s, 12H), 1.75 (m, 3H), 1.85-2.00 (m, 3H), 2.05 (m, 2H), 2.15-2.29 (m, 6H), 2.98 (m, 1H), 3.03-3.11 (m, 7H),

3.34 (m, 4H), 3.42 (m, 2H), 3.46 (m, 2H), 4.08 (m, 3H), 4.16 (m, 3H), 6.53 (m, 3H), 6.79 (s, 1H), 7.41 (m, 2H), 7.48 (m, 1H), 7.53 (m, 2H), 7.63-7.76 (m, 9H), 7.80-7.85 (m, 4H), 7.91 (m, 2H), 8.12 (m, 1H), 8.33 (m, 3H); MS (MALDI-TOF) m/z for $[C_{76}H_{91}BrClN_7O_{20}PS_2]^-$ calcd. 1630.4375, found 1630.2861.

RXP470-PEG$_2$-ZW800-1*,* **3**. A green solid was obtained (49%). In PBS: $\varepsilon_{279\ nm}$ = 29,500 $M^{-1}cm^{-1}$, $\varepsilon_{770\ nm}$= 233,200 $M^{-1}cm^{-1}$; In PBS, $\lambda_{ex}/\lambda_{em}$ = 772/785 nm; Rt = 5.94 min (Supelco Ascentis® Express C18 column, 0 to 100% B in 10 min) and Rt = 6.61 min (Grace Vision HT C18 HL column, 0 to 100% B in 10 min); ^{1}H NMR (600 MHz, d_6-DMSO) δ; 1.28 (s, 2H), 1.30 (s, 15H), 1.55 (m, 6H), 1.75-2.10 (m, 9H), 2.14 (m, 6H), 2.28 (m, 7H), 2.75 (m, 6H), 3.05 (m, 4H), 3.10 (s, 18H), 3.40 (m, 1H), 3.45 (m, 6H), 4.08 (m, 1H), 4.17 (m, 6H), 6.19 (m, 2H), 6.76 (s, 1H), 7.04 (m, 2H), 7.24 (m, 2H), 7.36 (m, 2H), 7.48 (m, 1H), 7.53 (m, 2H), 7.64 (m, 6H), 7.69 (m, 2H), 7.72 (m, 1H), 7.78-7.91 (m, 9H), 8.46 (bs, 1H); MS (MALDI-TOF) m/z for $[C_{96}H_{119}BrClN_9O_{21}PS_2]^-$ calcd. 1942.6577, found 1942.6816.

Control probe*,* **4**. A green solid was obtained (59%). In PBS: $\varepsilon_{230\ nm}$ = 37,300 $M^{-1}cm^{-1}$, $\varepsilon_{770\ nm}$= 212,500 $M^{-1}cm^{-1}$; $\lambda_{ex}/\lambda_{em}$ = 773/785 nm; Rt = 7.53 min (Supelco Ascentis® Express C18 column, 0 to 100% B in 10 min) and Rt = 6.70 min (Grace Vision HT C18 HL column, 0 to 100% B in 10 min); MS (ESI) m/z for $[C_{96}H_{123}BrIN_9O_{22}PS_2]^{2+}$ calcd 964,8, found 964.9.

*Stability of probes **3** and **4** in mouse blood***:** The probes (4 nmol) were incubated in blood (200 µL) with gentle agitation (Thermo mixer, 1000 rpm) at 37°C for 4 h. At 0, 2, and 4 h time points 40 µL of blood sample was collected and centrifuged at 4°C (3000 rpm, 20 min). 10 µL of the supernatant (plasma) was collected, and proteins were precipitated with methanol (90 µL). The sample was then vortexed for 30 s at room temperature and centrifuged at 4°C (3000 rpm, 15 min). 50 µL of the supernatant was analyzed by Liquid chromatography–mass spectrometry (LC-MS), UV detection was performed at the wavelength corresponding to the maximal absorbance of its conjugated fluorochrome (LC-MS, LC: Agilent XDB C18 column, 0 to 13 min: 5 to 70% B, 13 to 18 min: 70 to 100% B, MS: Electrospray positive mode of ionization).

Supplemental Figure 1. Probe stability in mouse blood at 37 °C for 4 hours. Representative RP-HPLC profiles at 772 nm after collection at different time points and mass spectrometry analyses in positive mode of ionisation for probe **3** (A) and control probe **4** (B).

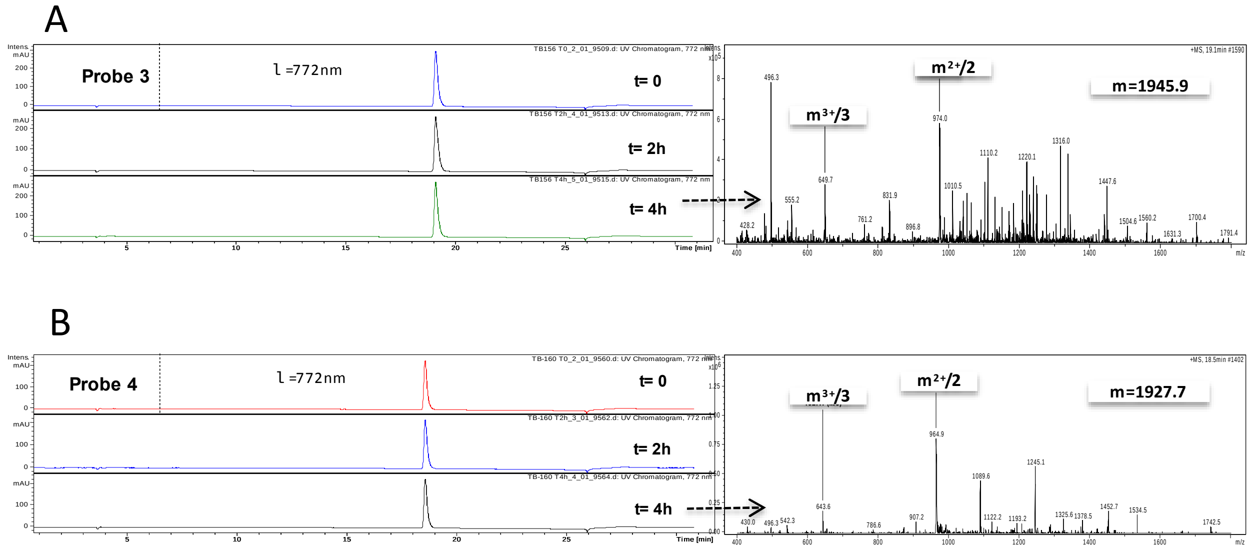

Supplemental Figure 2. Western blot analysis of MMP-2, -9, and -12 expression in sponge tissues harvested at day 1 and 4 after implantation.

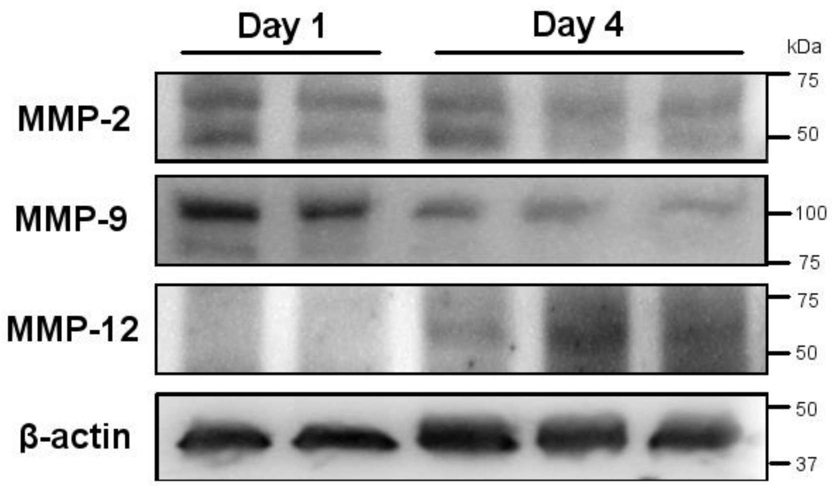

Supplemental Table 1. Calculated LogP (cLogP) and net charge values for RXP470.1, probes **1-3,** and their fluorophore moieties.

	Net charge of the compound	Net charge of the fluorophore moiety	cLogP[a]
RXP470.1	-3	-	3.67
Probe **1**	-6	-3	3.20
Probe **2**	-4	-1	3.79
Probe **3**	-2	+1	1.71

[a] The values were calculated using the online ALOGPS 2.1 program, compounds were considered under their non-protonated state.

Supplemental Table 2. K_i values (nM) for RXP470.1 and probes **1-4** towards a set of human (h) metalloproteases.

	**hMMP-1**	**hMMP-2**	**hMMP-3**	**hMMP-7**	**hMMP-8**	**hMMP-9**	**hMMP-10**	**hMMP-12**	**hMMP-13**	**hMMP-14**	**TACE**	**ADAMTS-5**
**RXP470.1**	>10000	52±4	80±2	518±40	120±40	170±50	17±20	**0.26±0.05**	11±10	150±21	>10000	6100±100
**1**	>10000	973±63	31±2	1623±13	2065±22	4167±41	71±50	**0.90±0.07**	74±70	1133±61	>10000	1490±129
**2**	7212±66	1068±18	188±22	1849±71	1439±35	8760±50	197±50	**6.1±0.1**	136±15	802±58	>10000	>10000
**3**	>10000	2127±172	367±61	4992±151	3309±348	4795±535	136±22	**3.4±0.3**	124±7	1019±69	4607±653	>10000
**4**	>10000	>12000	>12000	9800±320	7500±730	4903±1300	6430±1670	**4040±670**	7730±1530	7860±1530	>25000	>10000

Supplemental Table 3. Blood levels (in %injected dose per gram) of probes **3** and **4** in apoE$^{-/-}$ mice. n = 3-4 in each group.

Time (min)	Probe **3**	Probe **4**
30	2.7±0.1	2.4±0.3
60	2.3±0.3	2.0±0.2

^{1}H NMR spectrum of probe **1**

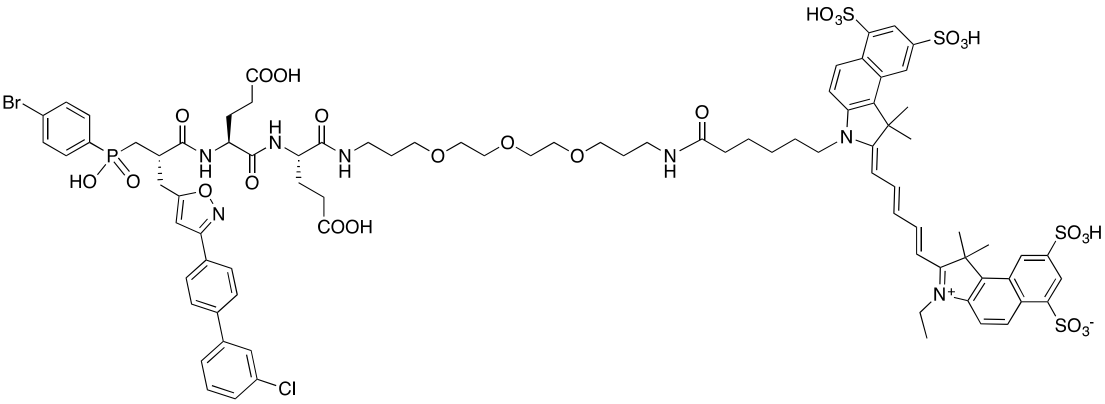

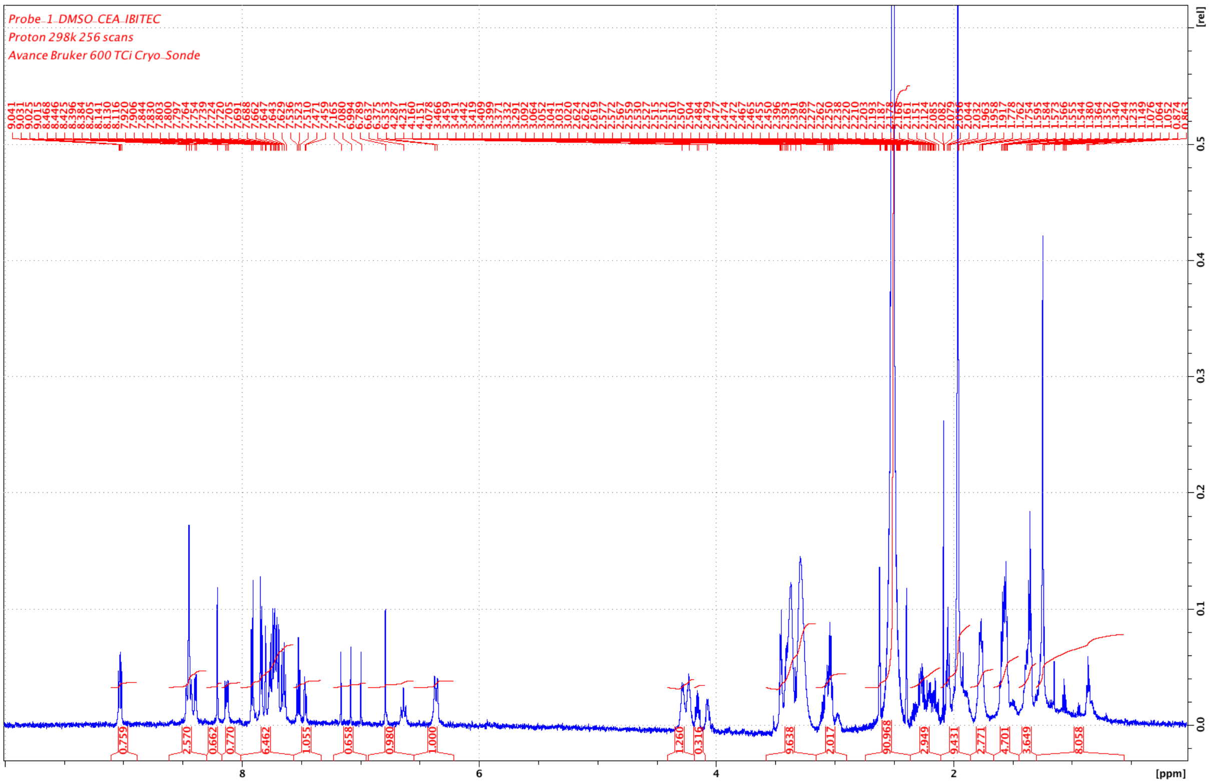

^{1}H NMR spectrum of probe **2**

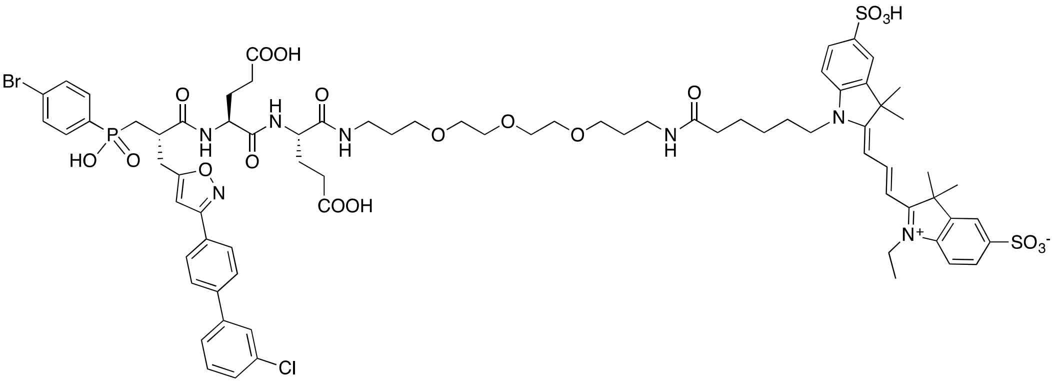

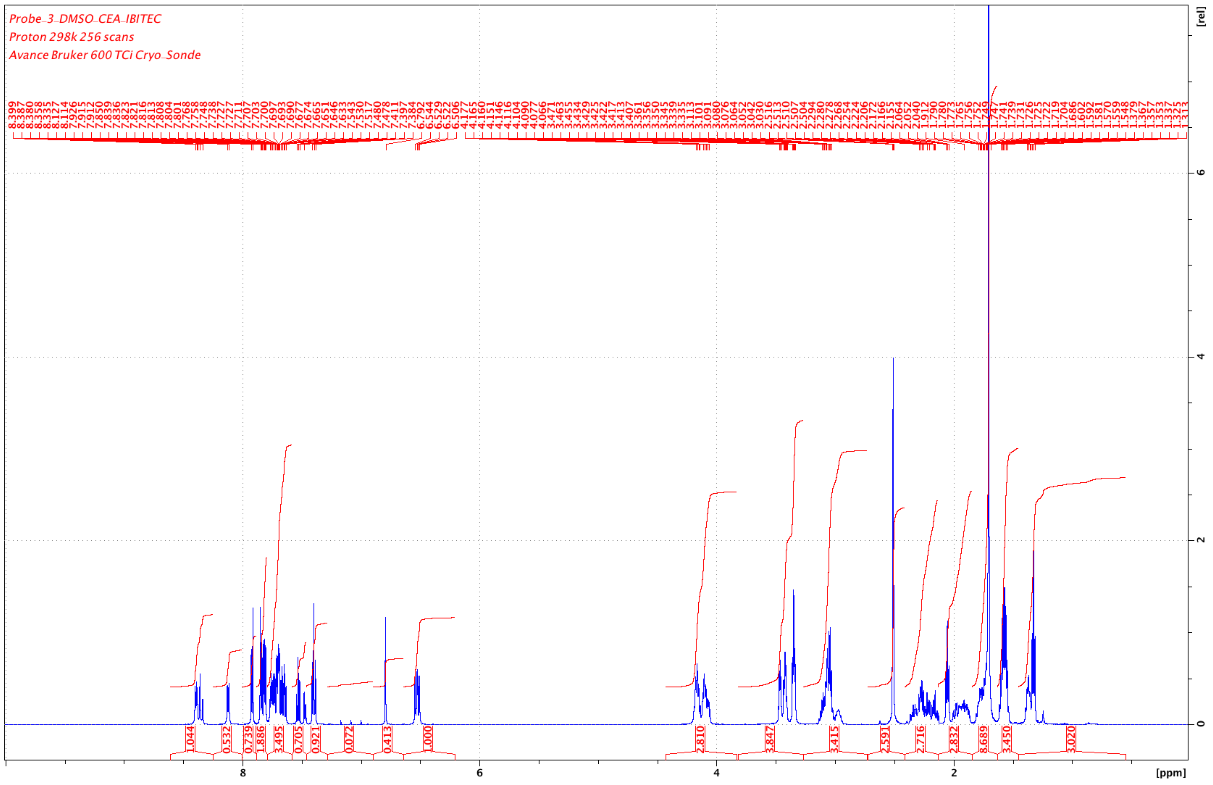

^{1}H NMR spectrum of probe **3**

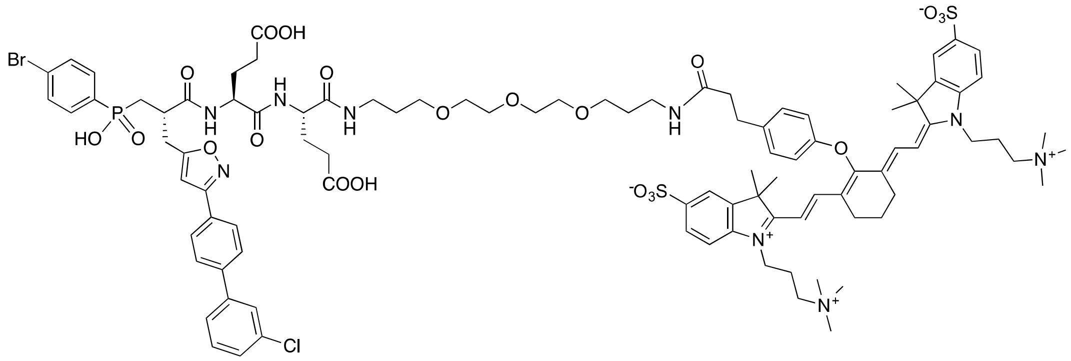

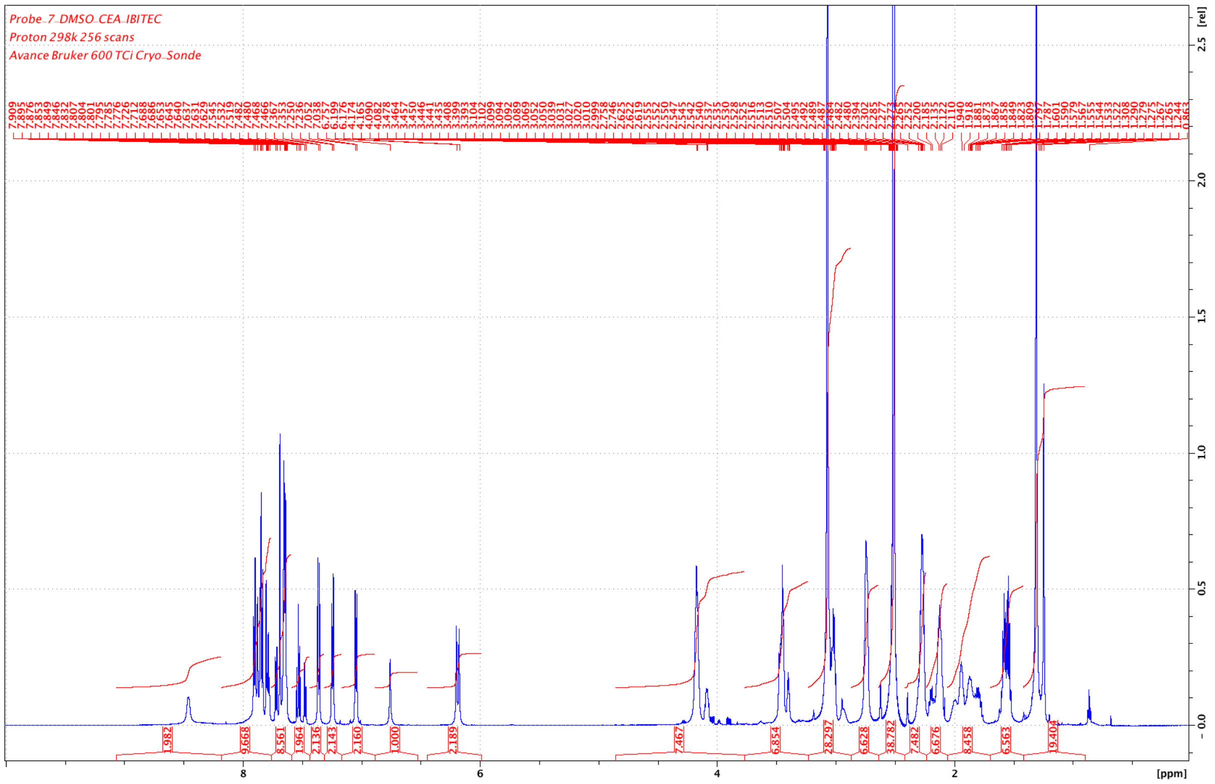

Supplement: Supplemental Data [file srep38345-s1.pdf]
